# T-box transcription factor TBX1, targeted by microRNA-6727-5p, inhibits cell growth and enhances cisplatin chemosensitivity of cervical cancer cells through AKT and MAPK pathways

**DOI:** 10.1080/21655979.2021.1880732

**Published:** 2021-02-08

**Authors:** Haixia Liu, Mei Song, Xiaoyan Sun, Xin Zhang, Huayan Miao, Yankui Wang

**Affiliations:** a Department of Gynecology, The Affiliated Hospital of Qingdao University, Qingdao, Shandong, P.R. China; b Department of Gynecology, The Third People’s Hospital of Qingdao, Qingdao, Shandong, P.R. China; c Department of Gynecology Oncology, The Affiliated Central Hospital of Qingdao University, Qingdao, Shandong, P.R. China

**Keywords:** Cervical cancer, t-box transcription factor 1, microRNA-6727-5p, akt, mapk

## Abstract

Cervical cancer (CC) is the fourth most common cancers among women worldwide. T-box transcription factor 1 (TBX1), a member of the T-box family, has anti-tumor effects in some types of cancer, but its role in CC is yet unknown. The aim of this study is to investigate the functions and underlying mechanisms of TBX1 in CC. Online database UALCAN showed that TBX1 was down-regulated in CC tissues compared with normal tissues and patients with lower TBX1 expression level had a poor prognosis. TBX1 overexpression significantly decreased the proliferation, migration, and invasion of Hela and SiHa cells. Conversely, cell apoptosis and chemosensitivity to cisplatin were promoted in TBX1-overexpressing CC cells. Moreover, up-regulation of TBX1 inhibited both AKT and MAPK signaling pathways. Furthermore, dual luciferase report assay indicated that TBX1 could directly bind to miR-6727-5p. In addition, TBX1 expression was inhibited by miR-6727-5p mimic and up-regulated by miR-6727-5p inhibitor. Knockdown of TBX1 reversed the inhibitory effect of the miR-6727-5p inhibitor on CC cells. This study demonstrates that TBX1, a target gene of miR-6727-5p, acts as a tumor suppressor in CC, indicating that TBX1 may be a new target for CC therapy.

## Graphical abstract

**Figure:**
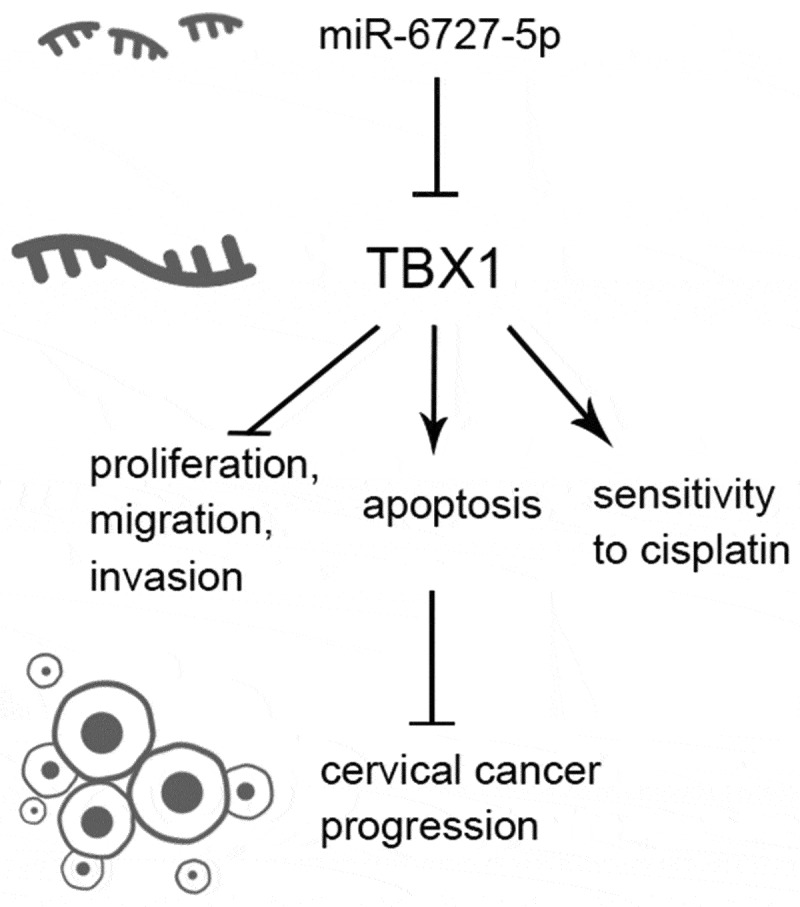

## Introduction

According to the 2018 global cancer data, cervical cancer (CC) ranks fourth for both incidence and mortality among women worldwide [1,2]. In China, the incidence of CC is increasing rapidly [3]. In the past two decades, in addition to surgical treatment, cisplatin-based concurrent chemotherapy along with adjuvant radiotherapy improved the overall survival (OS) and disease-free survival of patients with CC [4–6]. However, due to several reasons including cancer metastasis, recurrence, and drug resistance, the prognosis for patients remains poor [2,7]. Therefore, exploring new target for CC treatment is still of great significance.

T-box transcription factor 1 (TBX1) is a member of a phylogenetically conserved family of genes that share the common T-box DNA-binding domain [8]. TBX1 is the candidate gene for 22q11.2 microdeletion syndrome [9,10], and is also involved in heart disease, hypoparathyroidism, and acute kidney injury [11–13]. Additionally, aberrant expression of TBX1 has been detected in multiple types of cancer. For instance, TBX1 expression was down-regulated in parathyroid tumor [14] and thyroid cancer [15], while in basal cell carcinoma, TBX1 was highly expressed [16,17]. Besides, emerging studies have shown that TBX1 is a double-edged sword in the development of cancers. In thyroid cancer, TBX1 was identified as a tumor-suppressive gene [15,18]. Conversely, TBX1 exerted pro-oncogenic functions in parathyroid tumor and basal cell carcinoma [14,16]. However, the role of TBX1 in CC is still unknown.

MicroRNAs (miRs) are small noncoding RNAs that can target the 3ʹ untranslated region (3ʹUTR) of message RNAs (mRNAs) to degrade mRNAs and/or inhibit mRNA translation. Until now, several studies have reported the roles of miRs in cell proliferation, migration, invasion, and angiogenesis of CC [19–21]. Our previous study demonstrated that miR-6727-5p was highly expressed in CC tissues and promoted the proliferation of CC cells [22]. It was predicted by the TargetScan tool that the binding site of miR-6727-5p existed in the 3ʹUTR of TBX1. Therefore, TBX1 might be a potential target for miR-6727-5p in CC.

AKT and MAPK signal pathways participate in tumorigenic potential, cell cycle and cell apoptosis in CC [21,23–27]. TBX1 was also observed to inhibit tumor development by regulating the PI3K/AKT and MAPK/ERK signaling pathways in human thyroid cancer [15]. However, whether AKT and MAPK pathways are involved in the roles of TBX1 in CC has yet to be investigated.

In the present study, we aimed to investigate the effects and the underlying mechanism of TBX1 on the proliferation, migration, invasion, apoptosis, and cisplatin chemosensitivity of CC cells. In addition, we explored whether TBX1 was targeted and regulated by miR-6727-5p. This study provides novel insight into the molecular mechanism underlying CC.

## Materials and methods

### Biological information analysis

The expression and survival data of TBX1 in CC patients were obtained through the online database UALCAN (http://ualcan.path.uab.edu/index.html). UALCAN uses RNA-seq data from TCGA. Transcript per million (TPM) value was employed for estimating the expression level of TBX1. Box plot showed TBX1 expression level in normal and tumor samples, and the significance of difference between groups was estimated by Student’s t-test. Kaplan–Meier plot depicted the association of TBX1 expression levels with CC patient survival. The survival curves of samples with high TBX1 expression (with TPM values above 3rd quartile) and low/medium TBX1 expression (with TPM values below 3rd quartile) were compared by log rank test. A p value < 0.05 was considered as statistically significant [28].

### Cell culture and transfection

Human CC cells CaSki (Procell, China) were cultured in Roswell Park Memorial Institute-1640 Medium (RPMI-1640, Procell) supplemented with 10% fetal bovine serum (FBS, BioInd, Israel). Human CC cells SiHa (Procell) were cultured in Modified Eagle’s Medium (MEM, Procell) supplemented with 10% FBS. 293 T cells and human CC cells Hela (Shanghai Zhong Qiao Xin Zhou Biotechnology, China) were cultured in Dulbecco’s Modified Eagle’s Medium (DMEM, Shanghai Zhong Qiao Xin Zhou Biotechnology) supplemented with 10% FBS. Human cervical epithelial cells CerEpiC (Procell) were cultured in a special medium (Procell). All cells were maintained at 37°C with 5% CO_2_.

Cell transfection was performed using Lipofectamine^TM^2000 (Invitrogen, USA) according to the manufacturer’s protocol. TBX1 overexpression (OE-TBX1) plasmids and empty vectors were constructed by Wanlei Biotechnology Co., Ltd. (China) and transfected into SiHa and Hela cells for 48 hours. miR-6727-5p mimic, miR-6727-5p inhibitor, TBX1 siRNA and their negative control (NC) were also transfected into cells for 48 hours.

### Cell counting kit-8 (CCK-8) assay

To detect cell proliferation ability, cells (1 × 10^4^/well) were seeded in 96-well plates and transfected with OE-TBX1 plasmids, miR-6727-5p inhibitor, TBX1 siRNA or their negative control for 0, 24, 48, 72 and 96 hours. To test the cisplatin sensitivity of CC cells, cells (1 × 10^4^/well) were seeded in 96-well plates and transfected with OE-TBX1 plasmids or empty vectors for 24 hours, then treated with different concentrations of cisplatin (0, 2, 4, 8, 16, 32 and 64 μM; MeilunBio, China) for additional 24 hours. After the treatment, cells were incubated with CCK-8 regents (KeyGEN, China) for 2 hours, and the optical density (OD) at 450 nm was measured with a microplate reader (BIOTEK, USA).

### Flow cytometry

The apoptotic rate of cells was detected by flow cytometry using cell apoptosis detection kit (Beyotime, China). After plasmid transfection or cisplatin treatment, cells were digested, collected, and suspended with Annexin V-FITC binding buffer. Afterward, the cells were incubated with Annexin V-FITC and Propidium Iodide for 10 to 20 minutes in the dark room. Finally, the labeled cells were analyzed with a flow cytometer (Aceabio, USA).

### Transwell assay

Cell invasion was measured using 24-well transwell chambers (Corning, USA). After treatment, cells (1 × 10^4^/well) were seeded into the upper chamber pre-coated with Matrigel (BD, USA) in a serum-free medium and incubated for 48 hours. Medium with 10% FBS was added into the bottom chamber. The non-invading cells in the upper chambers were removed, and the invading cells in the bottom chambers were fixed with 4% paraformaldehyde (Aladdin, China) and stained with 0.4% purple crystal solution (Amresco, USA). Finally, the pictures were captured using an inverted microscope system (200× magnification, Olympus, Japan), and the number of invading cells was calculated.

### Wound-healing assay

SiHa and Hela cells were treated with a serum-free medium in the presence of mitomycin C (1 μg/ml, SIGMA, USA) for 1 hour. Then, the cells were transfected with OE-TBX1 plasmids or empty vectors and a 200-μl pipette tip was used to scratch the cells. Next, the cells were washed with a serum-free medium. The images of the wound were captured with the Olympus system (100× magnification, Olympus, Japan) at 0 and 48 hours and the wound closure was calculated.

### RNA extraction and quantitative real-time polymerase chain reaction (qRT-PCR)

Total RNA was extracted by using Total RNA extraction kit (Tiangen, China) according to the manufacturer’s protocol. The concentration of RNAs was measured using Nano 2000 (ThermoFisher, USA). The cDNA was synthesized using M-MLV reverse transcriptase (Tiangen). qRT-PCR was performed using SYBR Green (Solarbio, China) and 2× Taq PCR MasterMix (Tiangen) by Exicycler 96 RT-PCR instrument (BIONEER, Korea). The reaction conditions were conducted as follows: as for miRNA detection, pre-denaturation at 94°C for 2 min, a total of 40 cycles of denaturation at 94°C for 15 s, annealing at 60°C for 15 s, and extension at 72°C for 15 s; as for mRNA detection, pre-denaturation at 94°C for 5 min, a total of 40 cycles of denaturation at 94°C for 10 s, annealing at 60°C for 20 s, and extension at 72°C for 30 s. U6 and GAPDH served as internal controls for miRNA and mRNA, respectively. The 2^−ΔΔCt^ method was applied to calculate the relative expression of miR-6727-5p and TBX1. The details of primers (Genscript, China) are listed in Table 1.

**Table 1.** Primers utilized for qRT-PCR

Target	Primer sequence (5ʹ-3ʹ)
TBX1	Forward: CAACAACCTACTGGACGACAACG
	Reverse: CTCCTCGGCATATTTCTCGCTAT
GAPDH	Forward: GACCTGACCTGCCGTCTAG
	Reverse: AGGAGTGGGTGTCGCTGT
U6	Forward: GCTTCGGCAGCACATATACT
	Reverse: GTGCAGGGTCCGAGGTATTC
miR-6727-5p	Forward: ATCTCGGGGCAGGCGGCT
	Reverse: GCAGGGTCCGAGGTATTC

Note: qRT-PCR, quantitative real-time PCR; TBX1, T-box transcription factor 1; GAPDH, glyceraldehyde-3-phosphate dehydrogenase; miR-6727-5p, microRNA-6727-5p.

### Western blot

Total protein lysates from cells were separated by sodium dodecyl-sulfate polyacrylamide gel electrophoresis (SDS-PAGE) and transferred to polyvinylidene fluoride (PVDF, Millipore, USA) membranes. Then, the membranes were incubated with 5% nonfat milk (Sangon Biotech, China) in Tris-buffered saline with Tween 20 for 1 hour to inhibit nonspecific binding. The membranes were incubated with primary antibodies at 4°C overnight and then incubated with secondary antibodies at 37°C for 1 hour. Finally, the immunoreactive proteins were detected using electrochemiluminescence (ECL, Solarbio, China) and visualized using Gel-Pro-Analyzer. The information on antibodies is listed in Table 2.

**Table 2.** Antibodies utilized for Western blot

Antibody name	Dilution	Manufacturer	Cat
p-AKT (Ser473)	1:1000	CST	#4060
AKT	1:2000	CST	#4691
p-ERK1/2 (Thr202/Tyr204)	1:1000	Affinity	AF1015
ERK1/2	1:1000	Affinity	AF0155
p-p38 (Thr180/Tyr182)	1:500	Affinity	AF4001
p38	1:500	Affinity	AF6456
p-JNK (Thr183+ Tyr185)	1:1000	Affinity	AF3318
JNK	1:2000	Affinity	AF6318
TBX1	1:1000	Affinity	AF0327
GAPDH	1:10,000	Proteintech	60,004-1-Ig
Goat anti rabbit IgG-HRP	1:3000	Solarbio	SE134
Goat anti mouse IgG-HRP	1:3000	Solarbio	SE131

Note: AKT, protein kinase B; ERK, extracellular signal-regulated kinase; JNK, c-Jun NH2-terminal kinase; TBX1, T-box transcription factor 1; GAPDH, glyceraldehyde-3-phosphate dehydrogenase; IgG, immunoglobulin G; HRP, horseradish peroxidase.

### Dual luciferase report assay

The potential binding sites between miR-6727-5p and TBX1 were predicted through the online prediction database TargetScan (http://www.targetscan.org/vert_71/). The 3ʹUTR of TBX1 containing miR-6727-5p binding sequences was constructed into luciferase vectors to generate wt-TBX1 reporters, while 3ʹUTR of TBX1 containing the mutated miR-6727-5p binding sequences was constructed into luciferase vectors to generate mut-TBX1 reporters. The wt-TBX1 or mut-TBX1 was co-transfected with miR-6727-5p mimics or NC mimics into 293 T cells for 24 hours. Then, luciferase activities were detected by using dual luciferase reporter gene assay kits (KeyGEN, China) according to the manufacturer’s instructions. Renilla luciferase served as an internal control for normalization.

### Quantification and statistical analysis

Each experiment was performed at least three times, and the data were expressed as mean ± standard deviation (SD). All statistical analyses were performed using GraphPad Prism (v8.0). One-way ANOVA and Tukey’s multiple comparison test were used to measure the differences among groups. Two-way ANOVA was used to assess the statistical significance among groups tested at different times. A value of p < 0.05 was considered statistically significant.

## Results

In the present study, we hypothesized that TBX1 played an anti-tumor role in CC and was targeted by miR-6727-5p. At first, we analyzed the expression level of TBX1 in CC tissues and cell lines, and evaluated the correlation between TBX1 expression level and survival of CC patients. To investigate the function of TBX1 in CC, we tested the effects of TBX1 up-regulation on CC cell growth, metastasis, and chemosensitivity to cisplatin. In addition, we verified the target binding between TBX1 and miR-6727-5p, and further explored the role of TBX1 in miR-6727-5p-mediated tumorigenesis and metastasis in CC.

## The expression of TBX1 in CC tissues and cell lines

**Figure 1.**
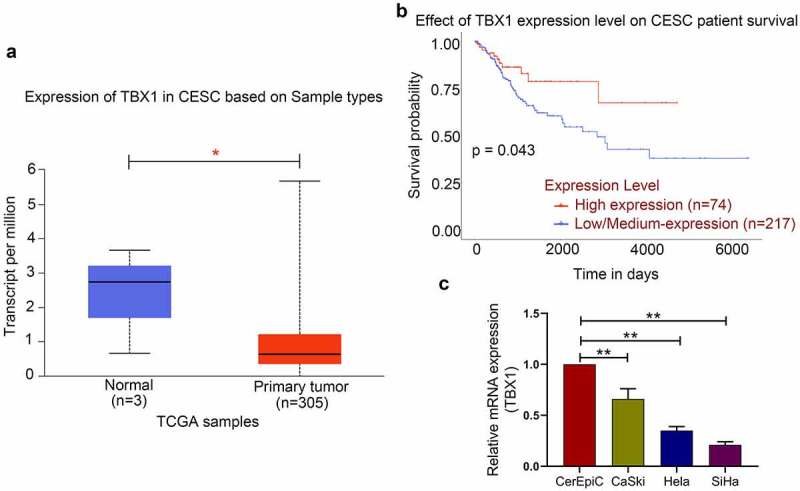
**TBX1 expression was decreased in CC tissues and cell lines**. (a) TBX1 mRNA expression in CESC tissues and normal tissues. (b) Kaplan-Meier plot of TBX1 in patients with CESC. (c) Relative mRNA expression of TBX1 was detected by qRT-PCR. CESC, cervical squamous cell carcinoma. * p < 0.05, ** p < 0.01

TBX1 expression in CC tissue and the survival analysis of its high or low/medium expression level in CC patients were obtained from the UALCAN database. The results showed that the expression level of TBX1 in CC tissues was significantly lower than that in normal tissues (Figure 1a) and patients with lower TBX1 expression level had poorer OS (Figure 1b). Then we detected the relative mRNA expression level of TBX1 in CerEpic and three CC cell lines (CaSki, Hela and SiHa) by qRT-PCR. We found that compared with the CerEpic, the relative mRNA expression level of TBX1 was decreased in three CC cell lines (CaSki: 0.66 ± 0.10, Hela: 0.35 ± 0.04, SiHa: 0.21 ± 0.03 *vs*. CerEpic: 1.00 ± 0.00; Figure 1c), which was consistent with the TBX1 expression in CC tissues. SiHa and Hela cells with lower TBX1 expression were selected for the following experiments.

## Effect of TBX1 overexpression on the growth and metastasis of CC cells

**Figure 2.**
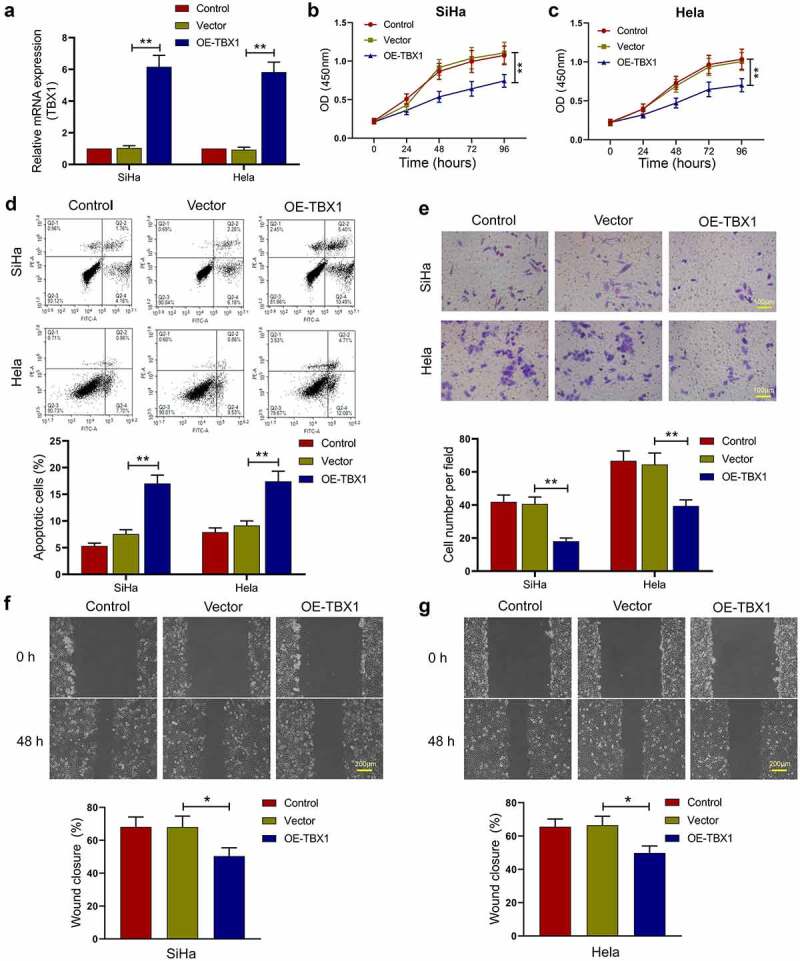
**TBX1 inhibited CC cell growth and metastasis**. (a) Relative mRNA expression of TBX1 was detected by qRT-PCR. (b, c) Cell viability was determined by CCK-8 assay. (d) Flow cytometry was used to detect the apoptosis rate of CC cells. (e) Cell invasion was detected by transwell assay. Scale bars: 100 μm. (f, g) Wound-healing assay was performed to determine cell migration ability. Scale bars: 200 μm. * p < 0.05, ** p < 0.01

To determine the role of TBX1 in the proliferation, migration, invasion, and apoptosis of CC cells, the TBX1 overexpression plasmids or empty vectors were transfected into SiHa and Hela cells, and the relative expression level of TBX1 mRNA was detected by qRT-PCR (SiHa OE-TBX1 *vs*. Vector: 6.16 ± 0.72 *vs*. 1.04 ± 0.14; Hela OE-TBX1 *vs*. Vector 5.83 ± 0.63 *vs*. 0.93 ± 0.16; Figure 2a). Then, CCK-8 assay was performed to measure the cell viability at 0, 24, 48, 72 and 96 hours after transfection. TBX1 overexpression dramatically inhibited the proliferation of CC cells (Figure 2b,C). In addition, flow cytometry analysis confirmed that TBX1 overexpression significantly increased the apoptotic rate of CC cells (SiHa OE-TBX1 *vs*. Vector: 17.03%±1.56% *vs*. 7.56%±0.80%; Hela OE-TBX1 *vs*. Vector: 17.42%±1.90% *vs*. 9.16%±0.84%; Figure 2d). On the basis of transwell assay and wound-healing assay, TBX1 overexpression significantly inhibited the invasion (SiHa OE-TBX1 *vs*. Vector: 18.13 ± 1.92 *vs*. 40.60 ± 4.26; Hela OE-TBX1 *vs*. Vector: 39.40 ± 3.67 *vs*. 64.47 ± 6.96; Figure 2e) and migration (SiHa OE-TBX1 *vs*. Vector: 50.37%±5.03% *vs*. 68.04%±6.64%; Hela OE-TBX1 *vs*. Vector: 49.83%±4.17% *vs*. 66.49%±5.38%; figure 2f, g) of CC cells. In general, these findings suggested that TBX1 inhibited CC cell proliferation, migration, and invasion, and promoted cell apoptosis.

## Effect of TBX1 overexpression on the chemosensitivity of CC cells to cisplatin

**Figure 3.**
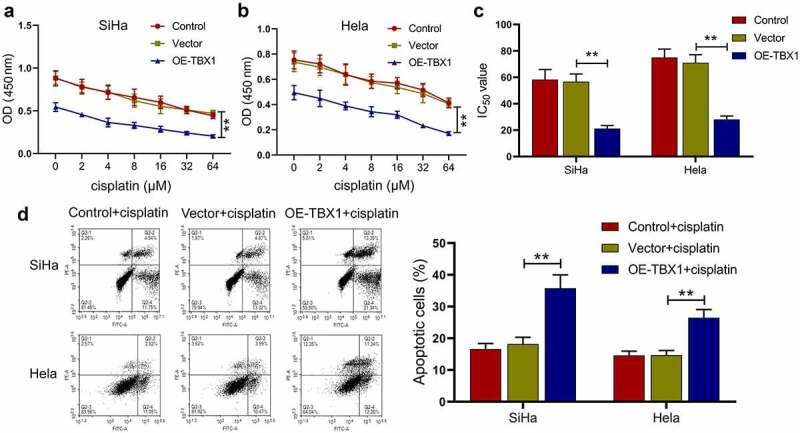
**TBX1 sensitized CC cells to cisplatin**. (a, b) CCK-8 assay was used to detect cell viability. (c) IC_50_ value was calculated to analyze the cisplatin chemosensitivity of CC cells. (d) CC cell apoptosis in the presence of cisplatin (5 μM and 7.5 μM, respectively) for 24 hours was detected by flow cytometry. ** p < 0.01

Next, it was investigated whether TBX1 overexpression could affect the sensitivity of CC cells to cisplatin. After transfection, SiHa and Hela cells were treated with different concentrations of cisplatin (0, 2, 4, 8, 16, 32 and 64 μM) and the cell viability was detected by CCK-8 assay. TBX1 overexpression was observed to decrease the cell viability of CC cells in the presence of cisplatin (Figure 3a, b) and 50% inhibitory concentration (IC_50_) of cisplatin (SiHa OE-TBX1 *vs*. Vector: 21.17 ± 2.30 μM *vs*. 56.75 ± 5.81 μM; Hela OE-TBX1 *vs*. Vector: 28.10 ± 2.61 μM *vs*. 71.03 ± 6.09 μM; Figure 3c), indicating that TBX1 overexpression strongly increased the sensitivity of CC cells to cisplatin. Moreover, we confirmed that TBX1 overexpression markedly promoted the apoptosis induced by cisplatin in CC cells (SiHa OE-TBX1 *vs*. Vector: 35.75%±4.21% *vs*. 18.20%±2.11%; Hela OE-TBX1 *vs*. Vector: 26.46%±2.61% *vs*. 14.67%±1.49%; Figure 3d). The above results illuminated that TBX1 overexpression enhanced the chemosensitivity of CC cells to cisplatin.

## Effect of TBX1 overexpression on the AKT and MAPK signal pathways in CC cells

**Figure 4.**
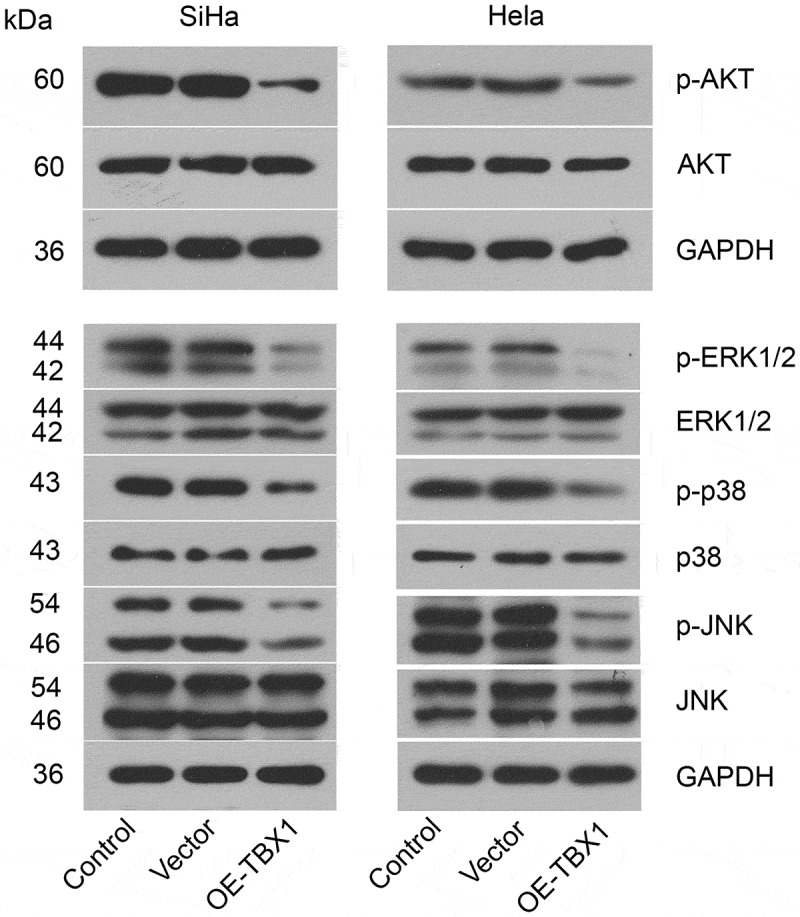
**TBX1 inactivated AKT and MAPK signal pathways in CC cells**. Western blot was used to evaluate the phosphorylation of proteins related to AKT and MAPK signal pathways in SiHa and Hela cells after transfection with OE-TBX1 plasmids or empty vectors

Subsequently, the impact of TBX1 on the AKT and MAPK signal pathways in CC cells was evaluated using Western blot analysis. As shown in Figure 4, TBX1 overexpression decreased the phosphorylation of AKT at Ser473, ERK1/2 at Thr202/Tyr204, p38 at Thr180/Tyr182 and JNK at Thr183/Tyr185, suggesting that AKT and MAPK signal pathways were involved in the role of TBX1 in CC cells.

## The relationship between miR-6727-5p and TBX1 in SiHa cells

**Figure 5.**
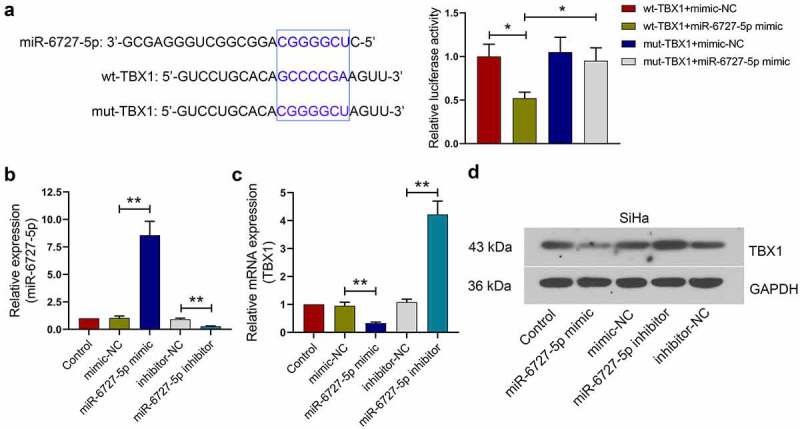
**miR-6727-5p targeted and decreased TBX1 expression in SiHa cells**. (a) Potential binding site between miR-6727-5p and TBX1 was predicted using TargetScan database and validated by dual luciferase reporter assay. (b, c) Relative expression of miR-6727-5p and TBX1 mRNA were detected by qRT-PCR. (d) Expression level of TBX1 protein was detected in SiHa cells by Western blot. * p < 0.05, ** p < 0.01

TargetScan database was used to predict the miR-6727-5p binding site in TBX1, and their binding was verified by using dual luciferase report assay. The relative luciferase activity was significantly decreased in 293 T cells co-transfected with miR-6727-5p mimic and wt-TBX1 plasmids, implying that TBX1 could bind to miR-6727-5p (wt-TBX1+ miR-6727-5p mimic *vs*. wt-TBX1+ mimic-NC, mut-TBX1+ miR-6727-5p mimic: 0.52 ± 0.07 *vs*. 1.00 ± 0.14, 0.95 ± 0.15; Figure 5a). Next, the miR-6727-5p mimic or miR-6727-5p inhibitor was transfected into CC cells, and the relative expression level of miR-6727-5p was tested by qRT-PCR (miR-6727-5p mimic *vs*. mimic-NC: 8.57 ± 1.26 *vs*. 1.03 ± 0.18; miR-6727-5p inhibitor *vs*. inhibitor-NC: 0.28 ± 0.03 *vs*. 0.91 ± 0.11; Figure 5b). The relative expression levels of TBX1 mRNA and protein in SiHa cells were decreased by miR-6727-5p mimic and increased by miR-6727-5p inhibitor (miR-6727-5p mimic *vs*. mimic-NC: 0.33 ± 0.04 *vs*. 0.95 ± 0.13; miR-6727-5p inhibitor *vs*. inhibitor-NC: 4.22 ± 0.48 *vs*. 1.08 ± 0.11; Figure 5c, d). These results suggested that TBX1 was directly targeted and down-regulated by miR-6727-5p in SiHa cells.

## Effect of TBX1 silencing on the anti-tumor role of miR-6727-5p inhibitor in SiHa cells

**Figure 6.**
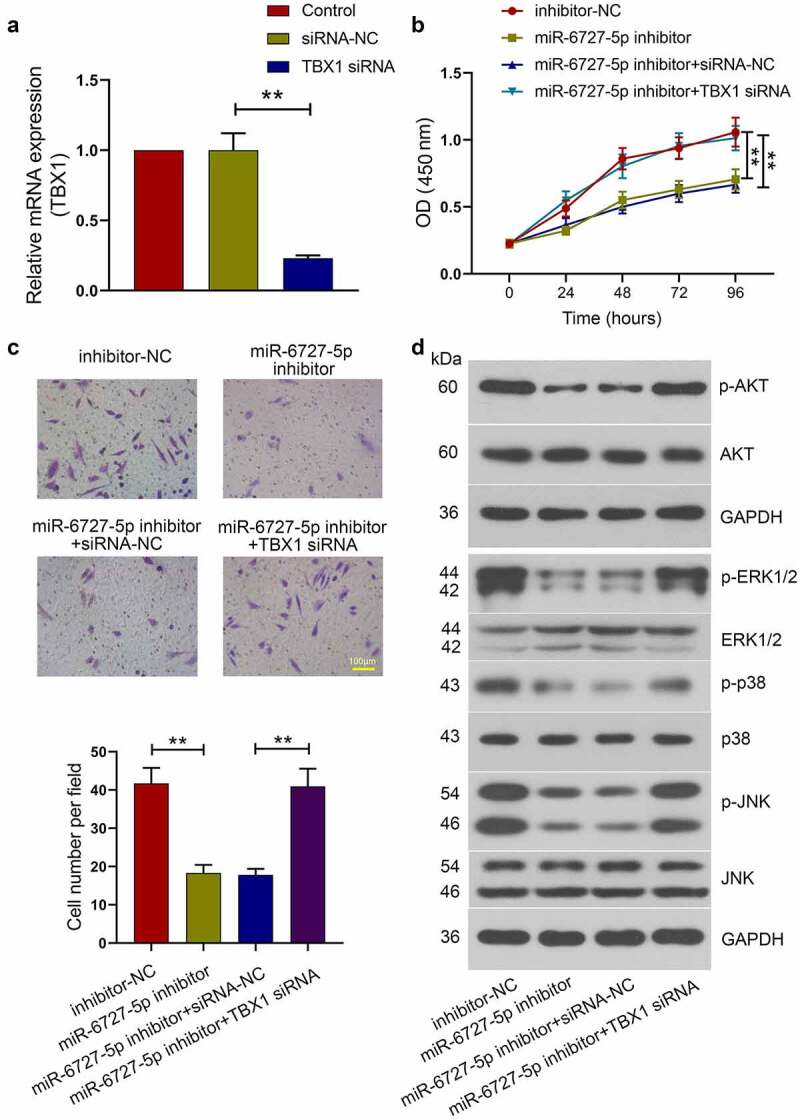
**TBX1 silencing reversed the effect of miR-6727-5p inhibitor on SiHa cells**. (a) Relative expression level of TBX1 mRNA was detected by qRT-PCR. (b) CCK-8 assay was used to measure cell viability. (c) Transwell assay was used to detect the invasion capacity of SiHa cells. Scale bar: 100 μm. (d) Western blot was adopted to detect the phosphorylation of proteins related to AKT and MAPK signal pathways. ** p < 0.01

To explore the role of TBX1 in miR-6727-5p-mediated tumorigenesis and metastasis in CC, miR-6727-5p inhibitor or inhibitor-NC was co-transfected with TBX1 siRNA or siRNA-NC into SiHa cells. The relative expression level of TBX1 mRNA was detected using qRT-PCR (TBX1 siRNA *vs*. siRNA-NC: 0.23 ± 0.02 *vs*. 1.00 ± 0.12; Figure 6a). CCK-8 assay showed that the viability of SiHa cells was decreased by miR-6727-5p inhibitor and the decrease was reversed by TBX1 silencing (Figure 6b). Transwell assay showed that the invasion of SiHa cells was inhibited by miR-6727-5p inhibitor and the inhibition was blocked by TBX1 silencing (miR-6727-5p inhibitor *vs*. inhibitor-NC: 18.33 ± 2.10 *vs*. 41.73 ± 4.02; miR-6727-5p inhibitor+TBX1 siRNA *vs*. miR-6727-5p inhibitor+siRNA-NC: 40.93 ± 4.65 *vs*. 17.80 ± 1.60; Figure 6c). Moreover, the inhibition of AKT, ERK, p38 and JNK phosphorylation induced by miR-6727-5p inhibitor was also reversed by knockdown of TBX1 (Figure 6d). These findings revealed that miR-6727-5p regulated the proliferation and invasion of SiHa cells by targeting TBX1.

## Discussion

In the present study, we found that TXB1 was down-regulated in CC tissues and cells, and low expression of TBX1 contributed to the poor OS of CC patients. We confirmed that TBX1 dramatically inhibited CC cell proliferation, migration and invasion and the activation of AKT and MAPK signaling, as well as promoted cell apoptosis and the sensitivity of CC cells to cisplatin. Moreover, TBX1 was directly targeted by miR-6727-5p and involved in the mechanism underlying the regulation of CC cell proliferation and invasion by miR-6727-5p.

TBX1 plays essential roles in embryonic development processes and regulates gene expression through epigenetic modifications [8]. However, its expression levels and functions varied in different types of tumor. In bladder urothelial carcinoma, TBX1 was up-regulated and contributed to a better prognosis of patients [29], while in the parathyroid tumor, TBX1-expressing cells were markedly reduced, and TBX1 deficiency potentially contributed to the low proliferative nature of tumors [14]. In the present study, TBX1 expression was decreased in CC tissues and cells, and low expression of TBX1 was correlated with the poor prognosis of CC patients, indicating that TBX1 might participate in the CC progression.

Emerging studies have reported the function of TBX1 in tumor progress. In thyroid cancer, the up-regulation of TBX1 remarkably inhibited cell proliferation and metastasis and tumorigenic potential in mice, and promoted cell apoptosis [15]. Similar results were observed in skin tumor of mice, in which TBX1 inhibited tumor growth and multilayered colony formation and induced cell cycle arrest [18]. Similar to previous studies, we found that TBX1 overexpression not only inhibited the cell growth, but also inhibited the migration and invasion abilities of CC cells, as well as promoted the apoptosis of CC cells.

Chemotherapy is a standard treatment for advanced CC and an adjuvant therapy after surgical resections [4]. A variety of molecules have been involved in chemoresistance, however, it remains unclear whether TBX1 participates in chemoresistance of CC. Given that chemoresistance had become the main obstacle for successful chemotherapy, we also determined whether TBX1 affected the chemosensitivity of CC to cisplatin. Notably, TBX1 overexpression dramatically enhanced the chemosensitivity of CC cells to cisplatin, indicating that TBX1 might be a new target for increasing the cisplatin sensitivity of CC patients. Taken together, although the specific role of TBX1 varied in different types of tumor, its anti-tumor effect on CC progression was strongly supported.

Besides, we explored the signal pathway involved in the role of TBX1 in CC. The functions of AKT and MAPK signal pathways in tumorigenesis were well studied [30,31]. Activation of AKT and MAPK signal pathways was reported to promote tumor growth and metastasis of CC [32]. In addition, blockade of AKT [27] or MAPK [26] signal pathway significantly attenuated the proliferation and migration activities of CC cells. Moreover, inhibition of the AKT pathway also induced apoptosis of CC cells [23–25]. In thyroid cancer, TBX1 exerted its tumor suppressor function through inhibiting phosphorylation of AKT and ERK [15]. In the present study, we observed inhibited phosphorylation of proteins related to AKT and MAPK pathways in TBX1-overexpressing CC cells, suggesting that TBX1 might exhibit inhibitory effects on CC cells through inactivation of AKT and MAPK signal pathways.

Accumulating evidences showed that miRs could participate in CC progression and metastasis. Down-regulation of miR-205 was reported to inhibit cell invasion and angiogenesis of CC through the AKT signaling pathway [21]. miR-99b was also revealed to suppress CC cell activity by inhibiting the PI3K/AKT/mTOR signaling pathway [33]. In our previous study, miR-6727-5p was up-regulated in CC and significantly promoted cell proliferation and migration, as well as suppressed the apoptosis of CC cells [22].

In the present study, we confirmed that TBX1 was targeted and down-regulated by miR-6727-5p in CC cells, and the silence of TBX1 reversed the anti-tumor effects of the miR-6727-5p inhibitor on CC cells. The findings indicated that decreased expression level of TBX1 at least partly resulted from miR-6727-5p up-regulation, therefore promoting the development and progression of CC. In a subsequent experiment, *in vivo* experiments need to be performed to further confirm the inhibitory effect of TBX1 on CC tumor development and metastasis.

## Conclusion

In summary, we demonstrated that TBX1 played an anti-tumor role in CC cells and enhanced the chemosensitivity of CC cells to cisplatin. Moreover, TBX1 was directly targeted and down-regulated by miR-6727-5p. This study provided a novel anti-tumor target for CC treatment.

## Supplementary Material

Supplemental Material

## Highlights

- TBX1 is downregulated in CC tissues and indicated a poor prognosis.
- TBX1 inhibits cell growth and enhances cisplatin chemosensitivity of CC cells.
- TBX1 inhibits both AKT and MAPK signaling pathways.
- TBX1 expression was regulated by miR-6727-5p via directly binding to its 3′-UTR.
