# Supplementary material for: T-box transcription factor TBX1, targeted by microRNA-6727-5p, inhibits cell growth and enhances cisplatin chemosensitivity of cervical cancer cells through AKT and MAPK pathways
Source: Bioengineered. 2021 Feb 8;12(1):565–77. doi: 10.1080/21655979.2021.1880732 (PMC8806341; doi:10.1080/21655979.2021.1880732)
Supplement: Supplemental Material [file KBIE_A_1880732_SM4793.zip › 1880732/Revised Manuscriptclean.docx]

**T-box transcription factor** **TBX1, targeted by microRNA-6727-5p, inhibits cell growth and enhances cisplatin chemosensitivity of** **cervical cancer cells through** **AKT and MAPK pathways**

**Running Head: Anti-tumor effect of TBX1 in cervical cancer.**

Haixia Liu^1, 2^, Mei Song^3^, Xiaoyan Sun^2^, Xin Zhang^2^, Huayan Miao^2^, Yankui Wang^1,^ *

^1^Department of Gynecology, The Affiliated Hospital of Qingdao University, Qingdao, Shandong, P.R. China

^2^Department of Gynecology, The Third People’s Hospital of Qingdao, Qingdao, Shandong, P.R. China

^3^Department of Gynecology Oncology，The Affiliated Central Hospital of Qingdao University, Qingdao, Shandong, P.R. China

*Correspondence author: Yankui Wang, Department of Gynecology, The Affiliated Hospital of Qingdao University, 16# Jiangsu Road, Qingdao, 266000, Shandong, P.R. China

Tel: +86-532-89076295

Fax: +86-532-89076510

Email: qdwyk2020@163.com

**Abstract** Cervical cancer (CC) is the fourth most common cancers among women worldwide. T-box transcription factor 1 (TBX1), a member of T-box family, has anti-tumor effects in some types of cancer, but its role in CC is yet unknown. The aim of this study is to investigate the functions and underlying mechanisms of TBX1 in CC. Online database UALCAN showed that TBX1 was down-regulated in CC tissues compared with normal tissues and patients with lower TBX1 expression level had a poor prognosis. TBX1 overexpression significantly decreased the proliferation, migration, and invasion of Hela and SiHa cells. Conversely, cell apoptosis and chemosensitivity to cisplatin were promoted in TBX1-overexpressing CC cells. Moreover, up-regulation of TBX1 inhibited both AKT and MAPK signaling pathways. Furthermore, dual luciferase report assay indicated that TBX1 could directly bind to miR-6727-5p. In addition, TBX1 expression was inhibited by miR-6727-5p mimic and up-regulated by miR-6727-5p inhibitor. Knockdown of TBX1 reversed the inhibitory effect of miR-6727-5p inhibitor on CC cells. This study demonstrates that TBX1, a target gene of miR-6727-5p, acts as a tumor suppressor in CC, indicating that TBX1 may be a new target for CC therapy.

**Key words:** cervical cancer; T-box transcription factor 1; microRNA-6727-5p; AKT; MAPK

**Introduction**

According to the 2018 global cancer data, cervical cancer (CC) ranks fourth for both incidence and mortality among women worldwide (Bray et al. 2018; Cohen et al. 2019). In China, the incidence of CC is increasing rapidly (Zhao and Qiao 2019). In the past two decades, in addition to surgical treatment, cisplatin-based concurrent chemotherapy along with adjuvant radiotherapy improved the overall survival (OS) and disease-free survival of patients with CC (Duenas-Gonzalez and Gonzalez-Fierro 2019; Feng et al. 2020; Vale et al. 2010). However, due to several reasons including cancer metastasis, recurrence, and drug resistance, the prognosis for patients remains poor (Cohen et al. 2019; Feng et al. 2017). Therefore, exploring new target for CC treatment is still of great significance.

T-box transcription factor 1 (TBX1) is a member of a phylogenetically conserved family of genes that share the common T-box DNA-binding domain (Papaioannou 2014). TBX1 is the candidate gene for 22q11.2 microdeletion syndrome (McDonald-McGinn et al. 2015; Scambler 2010), and is also involved in heart disease, hypoparathyroidism, and acute kidney injury (Chen et al. 2014; Jiang et al. 2014; Li et al. 2018). Additionally, aberrant expression of TBX1 has been detected in multiple types of cancer. For instance, TBX1 expression was down-regulated in parathyroid tumor (Verdelli et al. 2017) and thyroid cancer (Wang et al. 2019), while in basal cell carcinoma, TBX1 was highly expressed (Caprio et al. 2020; Sun and Jiang 2018). Besides, emerging studies have shown that TBX1 is a double-edged sword in the development of cancers. In thyroid cancer, TBX1 was identified as a tumor-suppressive gene (Trempus et al. 2011; Wang et al. 2019). Conversely, TBX1 exerted pro-oncogenic functions in parathyroid tumor and basal cell carcinoma (Caprio et al. 2020; Verdelli et al. 2017). However, the role of TBX1 in CC is still unknown.

MicroRNAs (miRs) are small noncoding RNAs that can target the 3’ untranslated region (3’UTR) of message RNAs (mRNAs) to degrade mRNAs and/or inhibit mRNAs translation. Until now, several studies have reported the roles of miRs in cell proliferation, migration, invasion, and angiogenesis of CC (Deng et al. 2015; Li et al. 2020; Zhang et al. 2019). Our previous study demonstrated that miR-6727-5p was highly expressed in CC tissues and promoted the proliferation of CC cells (Liu et al. 2017). It was predicted by TargetScan tool that the binding site of miR-6727-5p existed in the 3’UTR of TBX1. Therefore, TBX1 might be a potential target for miR-6727-5p in CC.

AKT and MAPK signal pathways participate in tumorigenic potential, cell cycle and cell apoptosis in CC (Hu et al. 2007; Liu et al. 2009; Prasad et al. 2015; Qi et al. 2020; Wang et al. 2020; Zhang et al. 2019). TBX1 was also observed to inhibit tumor development by regulating the PI3K/AKT and MAPK/ERK signaling pathways in human thyroid cancer (Wang et al. 2019). However, whether AKT and MAPK pathways are involved in the roles of TBX1 in CC has yet to be investigated.

In the present study, we aimed to investigate the effects and the underlying mechanism of TBX1 on the proliferation, migration, invasion, apoptosis, and cisplatin chemosensitivity of CC cells. In addition, we explored whether TBX1 was targeted and regulated by miR-6727-5p. This study provides novel insight into the molecular mechanism underlying CC.

**Materials and methods**

**Biological information analysis**

The expression and survival data of TBX1 in CC patients were obtained through online database UALCAN (<http://ualcan.path.uab.edu/index.html>). UALCAN uses RNA-seq data from TCGA. Transcript per million (TPM) value was employed for estimating the expression level of TBX1. Box plot showed TBX1 expression level in normal and tumor samples, and the significance of difference between groups was estimated by Student’s t-test. Kaplan-Meier plot depicted the association of TBX1 expression levels with CC patient survival. The survival curves of samples with high TBX1 expression (with TPM values above 3rd quartile) and low/medium TBX1 expression (with TPM values below 3rd quartile) were compared by log rank test. A p value < 0.05 was considered as statistically significant (Chandrashekar et al. 2017).

**Cell culture and transfection**

Human CC cells CaSki (Procell, China) were cultured in Roswell Park Memorial Institute-1640 Medium (RPMI-1640, Procell) supplemented with 10% fetal bovine serum (FBS, BioInd, Israel). Human CC cells SiHa (Procell) were cultured in Modified Eagle's Medium (MEM, Procell) supplemented with 10% FBS. 293T cells and human CC cells Hela (Shanghai Zhong Qiao Xin Zhou Biotechnology, China) were cultured in Dulbecco's Modified Eagle's Medium (DMEM, Shanghai Zhong Qiao Xin Zhou Biotechnology) supplemented with 10% FBS. Human cervical epithelial cells CerEpiC (Procell) were cultured in special medium (Procell). All cells were maintained at 37°C with 5% CO_2_.

Cell transfection was performed using Lipofectamine^TM^2000 (Invitrogen, USA) according to the manufacturer’s protocol. TBX1 overexpression (OE-TBX1) plasmids and empty vectors were constructed by Wanlei Biotechnology Co., Ltd. (China) and transfected into SiHa and Hela cells for 48 hours. miR-6727-5p mimic, miR-6727-5p inhibitor, TBX1 siRNA and their negative control (NC) were also transfected into cells for 48 hours.

**Cell counting kit-8 (CCK-8) assay**

To detect cell proliferation ability, cells (1×10^4^/well) were seeded in 96-well plates and transfected with OE-TBX1 plasmids, miR-6727-5p inhibitor, TBX1 siRNA or their negative control for 0, 24, 48, 72 and 96 hours. To test the cisplatin sensitivity of CC cells, cells (1×10^4^/well) were seeded in 96-well plates and transfected with OE-TBX1 plasmids or empty vectors for 24 hours, then treated with different concentrations of cisplatin (0, 2, 4, 8, 16, 32 and 64 μM; MeilunBio, China) for additional 24 hours. After the treatment, cells were incubated with CCK-8 regents (KeyGEN, China) for 2 hours, and the optical density (OD) at 450 nm was measured with a microplate reader (BIOTEK, USA).

**Flow cytometry**

The apoptotic rate of cells was detected by flow cytometry using cell apoptosis detection kit (Beyotime, China). After plasmid transfection or cisplatin treatment, cells were digested, collected, and suspended with Annexin V-FITC binding buffer. Afterwards, the cells were incubated with Annexin V-FITC and Propidium Iodide for 10 to 20 minutes in the dark room. Finally, the labelled cells were analyzed with a flow cytometer (Aceabio, USA).

**Transwell assay**

Cell invasion was measured using 24-well transwell chambers (Corning, USA). After treatment, cells (1×10^4^/well) were seeded into the upper chamber pre-coated with Matrigel (BD, USA) in serum-free medium and incubated for 48 hours. Medium with 10% FBS was added into the bottom chamber. The non-invading cells in the upper chambers were removed, and the invading cells in the bottom chambers were fixed with 4% paraformaldehyde (Aladdin, China) and stained with 0.4% purple crystal solution (Amresco, USA). Finally, the pictures were captured using an inverted microscope system (200× magnification, Olympus, Japan), and the number of invading cells were calculated.

**Wound healing assay**

SiHa and Hela cells were treated with serum-free medium in the presence of mitomycin C (1 μg/ml, SIGMA, USA) for 1 hour. Then, the cells were transfected with OE-TBX1 plasmids or empty vectors and 200-μl pipette tip was used to scratch the cells. Next, the cells were washed with serum-free medium. The images of the wound were captured with Olympus system (100× magnification, Olympus, Japan) at 0 and 48 hours and the wound closure was calculated.

**RNA extraction and quantitative real-time polymerase chain reaction (****qRT-PCR)**

Total RNA was extracted by using Total RNA extraction kit (Tiangen, China) according to the manufacturer’s protocol. The concentration of RNAs was measured using Nano 2000 (ThermoFisher, USA). The cDNA was synthesized using M-MLV reverse transcriptase (Tiangen). qRT-PCR was performed using SYBR Green (Solarbio, China) and 2×Taq PCR MasterMix (Tiangen) by Exicycler 96 RT-PCR instrument (BIONEER, Korea). The reaction conditions were conducted as follows: as for miRNA detection, pre-denaturation at 94°C for 2 min, a total of 40 cycles of denaturation at 94°C for 15 s, annealing at 60°C for 15 s, and extension at 72°C for 15 s; as for mRNA detection, pre-denaturation at 94°C for 5 min, a total of 40 cycles of denaturation at 94°C for 10 s, annealing at 60°C for 20 s, and extension at 72°C for 30 s. U6 and GAPDH served as internal controls for miRNA and mRNA, respectively. The 2^−ΔΔCt^ method was applied to calculate the relative expression of miR-6727-5p and TBX1. The details of primers (Genscript, China) were listed in Table 1.

**Western blot**

Total protein lysates from cells were separated by sodium dodecyl-sulfate polyacrylamide gel electrophoresis (SDS-PAGE) and transferred to polyvinylidene fluoride (PVDF, Millipore, USA) membranes. Then, the membranes were incubated with 5% nonfat milk (Sangon Biotech, China) in Tris-buffered saline with Tween 20 for 1 hour to inhibit nonspecific binding. The membranes were incubated with primary antibodies at 4°C overnight and then incubated with secondary antibodies at 37°C for 1 hour. Finally, the immunoreactive proteins were detected using electrochemiluminescence (ECL, Solarbio, China) and visualized using Gel-Pro-Analyzer. The information of antibodies was listed in Table 2.

**Dual luciferase report assay**

The potential binding sites between miR-6727-5p and TBX1 were predicted through online prediction database TargetScan (<http://www.targetscan.org/vert_71/>). The 3’UTR of TBX1 containing miR-6727-5p binding sequences was constructed into luciferase vectors to generate wt-TBX1 reporters, while 3’UTR of TBX1 containing the mutated miR-6727-5p binding sequences was constructed into luciferase vectors to generate mut-TBX1 reporters. The wt-TBX1 or mut-TBX1 was co-transfected with miR-6727-5p mimics or NC mimics into 293T cells for 24 hours. Then luciferase activities were detected by using dual luciferase reporter gene assay kits (KeyGEN, China) according to the manufacturer’s instructions. Renilla luciferase served as an internal control for normalization.

**Quantification and** **statistical analysis**

Each experiment was performed at least three times, and the data were expressed as mean ± standard deviation (SD). All statistical analyses were performed using GraphPad Prism (v8.0). One-way ANOVA and Tukey's multiple comparisons test were used to measure the differences among groups. Two-way ANOVA was used to assess the statistical significance among groups tested at different time. A value of p < 0.05 was considered statistically significant.

**Results**

In the present study, we hypothesized that TBX1 played an anti-tumor role in CC and was targeted by miR-6727-5p. At first, we analyzed the expression level of TBX1 in CC tissues and cell lines, and evaluated the correlation between TBX1 expression level and survival of CC patients. To investigate the function of TBX1 in CC, we tested the effects of TBX1 up-regulation on CC cell growth, metastasis, and chemosensitivity to cisplatin. In addition, we verified the target binding between TBX1 and miR-6727-5p, and further explored the role of TBX1 in miR-6727-5p-mediated tumorigenesis and metastasis in CC.

**The expression of TBX1 in CC** **tissues and cell lines**

TBX1 expression in CC tissue and the survival analysis of its high or low/medium expression level in CC patients were obtained from the UALCAN database. The results showed that the expression level of TBX1 in CC tissues was significantly lower than that in normal tissues (Fig. 1A) and patients with lower TBX1 expression level had poorer OS (Fig. 1B). Then we detected the relative mRNA expression level of TBX1 in CerEpic and three CC cell lines (CaSki, Hela and SiHa) by qRT-PCR. We found that compared with the CerEpic, the relative mRNA expression level of TBX1 was decreased in three CC cell lines (CaSki: 0.66±0.10, Hela: 0.35±0.04, SiHa: 0.21±0.03 *vs.* CerEpic: 1.00±0.00; Fig. 1C), which was consistent with the TBX1 expression in CC tissues. SiHa and Hela cells with lower TBX1 expression were selected for the following experiments.

**Effect of TBX1 overexpression on the growth and metastasis of** **CC cells**

To determine the role of TBX1 in the proliferation, migration, invasion, and apoptosis of CC cells, the TBX1 overexpression plasmids or empty vectors were transfected into SiHa and Hela cells, and the relative expression level of TBX1 mRNA was detected by qRT-PCR (SiHa OE-TBX1 *vs.* Vector: 6.16±0.72 *vs.* 1.04±0.14; Hela OE-TBX1 *vs.* Vector 5.83±0.63 *vs.* 0.93±0.16; Fig. 2A). Then CCK-8 assay was performed to measure the cell viability at 0, 24, 48, 72 and 96 hours after transfection. TBX1 overexpression dramatically inhibited the proliferation of CC cells (Fig. 2B, C). In addition, flow cytometry analysis confirmed that TBX1 overexpression significantly increased the apoptotic rate of CC cells (SiHa OE-TBX1 *vs.* Vector: 17.03%±1.56% *vs.* 7.56%±0.80%; Hela OE-TBX1 *vs.* Vector: 17.42%±1.90% *vs.* 9.16%±0.84%; Fig. 2D). On the basis of transwell assay and wound-healing assay, TBX1 overexpression significantly inhibited the invasion (SiHa OE-TBX1 *vs.* Vector: 18.13±1.92 *vs.* 40.60±4.26; Hela OE-TBX1 *vs.* Vector: 39.40±3.67 *vs.* 64.47±6.96; Fig. 2E) and migration (SiHa OE-TBX1 *vs.* Vector: 50.37%±5.03% *vs.* 68.04%±6.64%; Hela OE-TBX1 *vs.* Vector: 49.83%±4.17% *vs.* 66.49%±5.38%; Fig. 2F, G) of CC cells. In general, these findings suggested that TBX1 inhibited CC cell proliferation, migration, and invasion, and promoted cell apoptosis.

**Effect of TBX1 overexpression on the chemosensitivity of CC cells to cisplatin**

Next, it was investigated whether TBX1 overexpression could affect the sensitivity of CC cells to cisplatin. After transfection, SiHa and Hela cells were treated with different concentrations of cisplatin (0, 2, 4, 8, 16, 32 and 64 μM) and the cell viability was detected by CCK-8 assay. TBX1 overexpression was observed to decrease the cell viability of CC cells in the presence of cisplatin (Fig. 3A, B) and 50% inhibitory concentration (IC_50_) of cisplatin (SiHa OE-TBX1 *vs.* Vector: 21.17±2.30 μM *vs.* 56.75±5.81 μM; Hela OE-TBX1 *vs.* Vector: 28.10±2.61 μM *vs.* 71.03±6.09 μM; Fig. 3C), indicating that TBX1 overexpression strongly increased the sensitivity of CC cells to cisplatin. Moreover, we confirmed that TBX1 overexpression markedly promoted the apoptosis induced by cisplatin in CC cells (SiHa OE-TBX1 *vs.* Vector: 35.75%±4.21% *vs.* 18.20%±2.11%; Hela OE-TBX1 *vs.* Vector: 26.46%±2.61% *vs.* 14.67%±1.49%; Fig. 3D). The above results illuminated that TBX1 overexpression enhanced the chemosensitivity of CC cells to cisplatin.

**Effect of TBX1 overexpression on** **the AKT and MAPK** **signal pathways in CC cells**

Subsequently, the impact of TBX1 on the AKT and MAPK signal pathways in CC cells was evaluated using Western blot analysis. As shown in Fig. 4, TBX1 overexpression decreased the phosphorylation of AKT at Ser473, ERK1/2 at Thr202/Tyr204, p38 at Thr180/Tyr182 and JNK at Thr183/Tyr185, suggesting that AKT and MAPK signal pathways were involved in the role of TBX1 in CC cells.

**The relationship between miR-6727-5p and TBX1 in SiHa cells**

TargetScan database was used to predict the miR-6727-5p binding site in TBX1, and their binding was verified by using dual luciferase report assay. The relative luciferase activity was significantly decreased in 293T cells co-transfected with miR-6727-5p mimic and wt-TBX1 plasmids, impling that TBX1 could bind to miR-6727-5p (wt-TBX1+miR-6727-5p mimic *vs.* wt-TBX1+ mimic-NC, mut-TBX1+miR-6727-5p mimic: 0.52±0.07 *vs.* 1.00±0.14, 0.95±0.15; Fig. 5A). Next, the miR-6727-5p mimic or miR-6727-5p inhibitor was transfected into CC cells, and the relative expression level of miR-6727-5p was tested by qRT-PCR (miR-6727-5p mimic *vs.* mimic-NC: 8.57±1.26 *vs.* 1.03±0.18; miR-6727-5p inhibitor *vs.* inhibitor-NC: 0.28±0.03 *vs.* 0.91±0.11; Fig. 5B). The relative expression levels of TBX1 mRNA and protein in SiHa cells were decreased by miR-6727-5p mimic and increased by miR-6727-5p inhibitor (miR-6727-5p mimic *vs.* mimic-NC: 0.33±0.04 *vs.* 0.95±0.13; miR-6727-5p inhibitor *vs.* inhibitor-NC: 4.22±0.48 *vs.* 1.08±0.11; Fig. 5C, D). These results suggested that TBX1 was directly targeted and down-regulated by miR-6727-5p in SiHa cells.

**Effect of TBX1 silencing on the** **anti-tumor role of** **miR-6727-5p inhibitor** **in SiHa cells**

To explore the role of TBX1 in miR-6727-5p-mediated tumorigenesis and metastasis in CC, miR-6727-5p inhibitor or inhibitor-NC was co-transfected with TBX1 siRNA or siRNA-NC into SiHa cells. The relative expression level of TBX1 mRNA was detected using qRT-PCR (TBX1 siRNA *vs.* siRNA*-*NC: 0.23±0.02 *vs.* 1.00±0.12; Fig. 6A). CCK-8 assay showed that the viability of SiHa cells was decreased by miR-6727-5p inhibitor and the decrease was reversed by TBX1 silencing (Fig. 6B). Transwell assay showed that the invasion of SiHa cells was inhibited by miR-6727-5p inhibitor and the inhibition was blocked by TBX1 silencing (miR-6727-5p inhibitor *vs.* inhibitor-NC: 18.33±2.10 *vs.* 41.73±4.02; miR-6727-5p inhibitor+TBX1 siRNA *vs.* miR-6727-5p inhibitor+siRNA-NC: 40.93±4.65 *vs.* 17.80±1.60; Fig. 6C). Moreover, the inhibition of AKT, ERK, p38 and JNK phosphorylation induced by miR-6727-5p inhibitor was also reversed by knockdown of TBX1 (Fig. 6D). These findings revealed that miR-6727-5p regulated the proliferation and invasion of SiHa cells by targeting TBX1.

**Discussion**

In the present study, we found that TXB1 was down-regulated in CC tissues and cells, and low expression of TBX1 contributed to the poor OS of CC patients. We confirmed that TBX1 dramatically inhibited CC cell proliferation, migration and invasion and the activation of AKT and MAPK signaling, as well as promoted cell apoptosis and the sensitivity of CC cells to cisplatin. Moreover, TBX1 was directly targeted by miR-6727-5p and involved in the mechanism underlying the regulation of CC cell proliferation and invasion by miR-6727-5p.

TBX1 plays essential roles in embryonic development processes and regulates gene expression through epigenetic modifications (Papaioannou 2014). However, its expression levels and functions varied in different types of tumor. In bladder urothelial carcinoma, TBX1 was up-regulated and contributed to a better prognosis of patients (Song et al. 2020), while in parathyroid tumor, TBX1-expressing cells were markedly reduced, and TBX1 deficiency potentially contributed to the low proliferative nature of tumors (Verdelli et al. 2017). In the present study, TBX1 expression was decreased in CC tissues and cells, and low expression of TBX1 was correlated with the poor prognosis of CC patients, indicating that TBX1 might participate in the CC progression.

Emerging studies have reported the function of TBX1 in tumor progress. In thyroid cancer, the up-regulation of TBX1 remarkably inhibited cell proliferation and metastasis and tumorigenic potential in mice, and promoted cell apoptosis (Wang et al. 2019). Similar results were observed in skin tumor of mice, in which TBX1 inhibited tumor growth and multilayered colonies formation and induced cell cycle arrest (Trempus et al. 2011). Similar to previous studies, we found that TBX1 overexpression not only inhibited the cell growth, but also inhibited the migration and invasion ability of CC cells, as well as promoted the apoptosis of CC cells.

Chemotherapy is a standard treatment for advanced CC and an adjuvant therapy after surgical resections (Duenas-Gonzalez and Gonzalez-Fierro 2019). A variety of molecules have been involved in chemoresistance, however, it remains unclear whether TBX1 participates in chemoresistance of CC. Given that chemoresistance had become the main obstacle for successful chemotherapy, we also determined whether TBX1 affected the chemosensitivity of CC to cisplatin. Notably, TBX1 overexpression dramatically enhanced the chemosensitivity of CC cells to cisplatin, indicating that TBX1 might be a new target for increasing the cisplatin sensitivity of CC patients. Taken together, although the specific role of TBX1 varied in different types of tumor, its anti-tumor effect on CC progression was strongly supported.

Besides, we explored the signal pathway involved in the role of TBX1 in CC. The functions of AKT and MAPK signal pathways in tumorigenesis were well studied (Gagliardi et al. 2018; Kim et al. 2020). Activation of AKT and MAPK signal pathways was reported to promote tumor growth and metastasis of CC (Che et al. 2019). In addition, blockade of AKT (Wang et al. 2020) or MAPK (Qi et al. 2020) signal pathway significantly attenuated the proliferation and migration activities of CC cells. Moreover, inhibition of AKT pathway also induced apoptosis of CC cells (Hu et al. 2007; Liu et al. 2009; Prasad et al. 2015). In thyroid cancer, TBX1 exerted its tumor suppressor function through inhibiting phosphorylation of AKT and ERK (Wang et al. 2019). In the present study, we observed inhibited phosphorylation of proteins related to AKT and MAPK pathways in TBX1-overexpressing CC cells, suggesting that TBX1 might exhibit inhibitory effects on CC cells through inactivation of AKT and MAPK signal pathways.

Accumulating evidences showed that miRs could participate in CC progression and metastasis. Down-regulation of miR-205 was reported to inhibit cell invasion and angiogenesis of CC through AKT signaling pathway (Zhang et al. 2019). miR-99b was also revealed to suppress CC cell activity by inhibiting the PI3K/AKT/mTOR signaling pathway (Li et al. 2019). In our previous study, miR-6727-5p was up-regulated in CC and significantly promoted cell proliferation and migration, as well as suppressed the apoptosis of CC cells (Liu et al. 2017).

In the present study, we confirmed that TBX1 was targeted and down-regulated by miR-6727-5p in CC cells, and the silence of TBX1 reversed the anti-tumor effects of miR-6727-5p inhibitor on CC cells. The findings indicated that decreased expression level of TBX1 at least partly resulted from miR-6727-5p up-regulation, therefore promoting the development and progression of CC. In subsequent experiment, *in vivo* experiments need to be performed to further confirm the inhibitory effect of TBX1 on CC tumor development and metastasis.

**Conclusion**

In summary, we demonstrated that TBX1 played an anti-tumor role in CC cells and enhanced the chemosensitivity of CC cells to cisplatin. Moreover, TBX1 was directly targeted and down-regulated by miR-6727-5p. This study provided a novel anti-tumor target for CC treatment.

**Conflicts Statement**

The authors declare no conflicts of interest.

**Funding statement**

The authors declare no specific funding for this work.

**References**

Bray, F., Ferlay, J., Soerjomataram, I., Siegel, R.L., Torre, L.A., and Jemal, A. 2018. Global cancer statistics 2018: GLOBOCAN estimates of incidence and mortality worldwide for 36 cancers in 185 countries. CA: a cancer journal for clinicians 68(6): 394-424. doi:10.3322/caac.21492.

Caprio, C., Varricchio, S., Bilio, M., Feo, F., Ferrentino, R., Russo, D., et al. 2020. TBX1 and Basal Cell Carcinoma: Expression and Interactions with Gli2 and Dvl2 Signaling. International journal of molecular sciences 21(2). doi:10.3390/ijms21020607.

Chandrashekar, D.S., Bashel, B., Balasubramanya, S.A.H., Creighton, C.J., Ponce-Rodriguez, I., Chakravarthi, B., et al. 2017. UALCAN: A Portal for Facilitating Tumor Subgroup Gene Expression and Survival Analyses. Neoplasia (New York, N.Y.) 19(8): 649-658. doi:10.1016/j.neo.2017.05.002.

Che, Y., Li, Y., Zheng, F., Zou, K., Li, Z., Chen, M., et al. 2019. TRIP4 promotes tumor growth and metastasis and regulates radiosensitivity of cervical cancer by activating MAPK, PI3K/AKT, and hTERT signaling. Cancer letters 452: 1-13. doi:10.1016/j.canlet.2019.03.017.

Chen, M., Yang, Y.S., Shih, J.C., Lin, W.H., Lee, D.J., Lin, Y.S., et al. 2014. Microdeletions/duplications involving TBX1 gene in fetuses with conotruncal heart defects which are negative for 22q11.2 deletion on fluorescence in-situ hybridization. Ultrasound in obstetrics & gynecology : the official journal of the International Society of Ultrasound in Obstetrics and Gynecology 43(4): 396-403. doi:10.1002/uog.12550.

Cohen, P.A., Jhingran, A., Oaknin, A., and Denny, L. 2019. Cervical cancer. Lancet (London, England) 393(10167): 169-182. doi:10.1016/s0140-6736(18)32470-x.

Deng, B., Zhang, Y., Zhang, S., Wen, F., Miao, Y., and Guo, K. 2015. MicroRNA-142-3p inhibits cell proliferation and invasion of cervical cancer cells by targeting FZD7. Tumour biology : the journal of the International Society for Oncodevelopmental Biology and Medicine 36(10): 8065-8073. doi:10.1007/s13277-015-3483-2.

Duenas-Gonzalez, A., and Gonzalez-Fierro, A. 2019. Pharmacodynamics of current and emerging treatments for cervical cancer. Expert opinion on drug metabolism & toxicology 15(8): 671-682. doi:10.1080/17425255.2019.1648431.

Feng, C.H., Mell, L.K., Sharabi, A.B., McHale, M., and Mayadev, J.S. 2020. Immunotherapy With Radiotherapy and Chemoradiotherapy for Cervical Cancer. Seminars in radiation oncology 30(4): 273-280. doi:10.1016/j.semradonc.2020.05.003.

Feng, Y., Zou, W., Hu, C., Li, G., Zhou, S., He, Y., et al. 2017. Modulation of CASC2/miR-21/PTEN pathway sensitizes cervical cancer to cisplatin. Archives of biochemistry and biophysics 623-624: 20-30. doi:10.1016/j.abb.2017.05.001.

Gagliardi, P.A., Puliafito, A., and Primo, L. 2018. PDK1: At the crossroad of cancer signaling pathways. Seminars in cancer biology 48: 27-35. doi:10.1016/j.semcancer.2017.04.014.

Hu, H.Z., Yang, Y.B., Xu, X.D., Shen, H.W., Shu, Y.M., Ren, Z., et al. 2007. Oridonin induces apoptosis via PI3K/Akt pathway in cervical carcinoma HeLa cell line. Acta pharmacologica Sinica 28(11): 1819-1826. doi:10.1111/j.1745-7254.2007.00667.x.

Jiang, H., Li, L., Li-Ling, J., Qiu, G., Niu, Z., Jiang, H., et al. 2014. Increased Tbx1 expression may play a role via TGFβ-Smad2/3 signaling pathway in acute kidney injury induced by gentamicin. International journal of clinical and experimental pathology 7(4): 1595-1605.

Kim, H.K., Bhattarai, K.R., Junjappa, R.P., Ahn, J.H., Pagire, S.H., Yoo, H.J., et al. 2020. TMBIM6/BI-1 contributes to cancer progression through assembly with mTORC2 and AKT activation. Nature communications 11(1): 4012. doi:10.1038/s41467-020-17802-4.

Li, C., Ding, D., Gao, Y., and Li, Y. 2020. MicroRNA-3651 promotes colorectal cancer cell proliferation through directly repressing T-box transcription factor 1. International journal of molecular medicine 45(3): 956-966. doi:10.3892/ijmm.2020.4458.

Li, D., Gordon, C.T., Oufadem, M., Amiel, J., Kanwar, H.S., Bakay, M., et al. 2018. Heterozygous Mutations in TBX1 as a Cause of Isolated Hypoparathyroidism. The Journal of clinical endocrinology and metabolism 103(11): 4023-4032. doi:10.1210/jc.2018-01260.

Li, Y.J., Wang, Y., and Wang, Y.Y. 2019. MicroRNA-99b suppresses human cervical cancer cell activity by inhibiting the PI3K/AKT/mTOR signaling pathway. Journal of cellular physiology 234(6): 9577-9591. doi:10.1002/jcp.27645.

Liu, H., Pei, G., Song, M., Dai, S., and Wang, Y. 2017. Influence of hsa-miR-6727-5p on the proliferation, apoptosis, invasion and migration of Caski, Hela and SiHa cervical cancer cells. Journal of B.U.ON. : official journal of the Balkan Union of Oncology 22(4): 973-978.

Liu, Y., Xing, H., Weng, D., Song, X., Qin, X., Xia, X., et al. 2009. Inhibition of Akt signaling by SN-38 induces apoptosis in cervical cancer. Cancer letters 274(1): 47-53. doi:10.1016/j.canlet.2008.08.037.

McDonald-McGinn, D.M., Sullivan, K.E., Marino, B., Philip, N., Swillen, A., Vorstman, J.A., et al. 2015. 22q11.2 deletion syndrome. Nature reviews. Disease primers 1: 15071. doi:10.1038/nrdp.2015.71.

Papaioannou, V.E. 2014. The T-box gene family: emerging roles in development, stem cells and cancer. Development (Cambridge, England) 141(20): 3819-3833. doi:10.1242/dev.104471.

Prasad, S.B., Yadav, S.S., Das, M., Modi, A., Kumari, S., Pandey, L.K., et al. 2015. PI3K/AKT pathway-mediated regulation of p27(Kip1) is associated with cell cycle arrest and apoptosis in cervical cancer. Cellular oncology (Dordrecht) 38(3): 215-225. doi:10.1007/s13402-015-0224-x.

Qi, Y.L., Li, Y., Man, X.X., Sui, H.Y., Zhao, X.L., Zhang, P.X., et al. 2020. CXCL3 overexpression promotes the tumorigenic potential of uterine cervical cancer cells via the MAPK/ERK pathway. Journal of cellular physiology 235(5): 4756-4765. doi:10.1002/jcp.29353.

Scambler, P.J. 2010. 22q11 deletion syndrome: a role for TBX1 in pharyngeal and cardiovascular development. Pediatric cardiology 31(3): 378-390. doi:10.1007/s00246-009-9613-0.

Song, Y., Jin, D., Chen, J., Luo, Z., Chen, G., Yang, Y., et al. 2020. Identification of an immune-related long non-coding RNA signature and nomogram as prognostic target for muscle-invasive bladder cancer. Aging 12(12): 12051-12073. doi:10.18632/aging.103369.

Sun, H., and Jiang, P. 2018. MicroRNA-451a acts as tumor suppressor in cutaneous basal cell carcinoma. Molecular genetics & genomic medicine 6(6): 1001-1009. doi:10.1002/mgg3.473.

Trempus, C.S., Wei, S.J., Humble, M.M., Dang, H., Bortner, C.D., Sifre, M.I., et al. 2011. A novel role for the T-box transcription factor Tbx1 as a negative regulator of tumor cell growth in mice. Molecular carcinogenesis 50(12): 981-991. doi:10.1002/mc.20768.

Vale, C.L., Tierney, J.F., Davidson, S.E., Drinkwater, K.J., and Symonds, P. 2010. Substantial improvement in UK cervical cancer survival with chemoradiotherapy: results of a Royal College of Radiologists' audit. Clinical oncology (Royal College of Radiologists (Great Britain)) 22(7): 590-601. doi:10.1016/j.clon.2010.06.002.

Verdelli, C., Avagliano, L., Guarnieri, V., Cetani, F., Ferrero, S., Vicentini, L., et al. 2017. Expression, function, and regulation of the embryonic transcription factor TBX1 in parathyroid tumors. Laboratory investigation; a journal of technical methods and pathology 97(12): 1488-1499. doi:10.1038/labinvest.2017.88.

Wang, N., Li, Y., Wei, J., Pu, J., Liu, R., Yang, Q., et al. 2019. TBX1 Functions as a Tumor Suppressor in Thyroid Cancer Through Inhibiting the Activities of the PI3K/AKT and MAPK/ERK Pathways. Thyroid : official journal of the American Thyroid Association 29(3): 378-394. doi:10.1089/thy.2018.0312.

Wang, Q., Yan, S.P., Chu, D.X., Xie, Y., Wang, C.F., Zhang, J.Y., et al. 2020. Silencing of Long Non-coding RNA RP1-93H18.6 Acts as a Tumor Suppressor in Cervical Cancer through the Blockade of the PI3K/Akt Axis. Molecular therapy. Nucleic acids 19: 304-317. doi:10.1016/j.omtn.2019.10.041.

Zhang, F., Liu, J., and Xie, B.B. 2019. Downregulation of microRNA-205 inhibits cell invasion and angiogenesis of cervical cancer through TSLC1-mediated Akt signaling pathway. Journal of cellular physiology 234(10): 18626-18638. doi:10.1002/jcp.28501.

Zhao, F., and Qiao, Y. 2019. Cervical cancer prevention in China: a key to cancer control. Lancet (London, England) 393(10175): 969-970. doi:10.1016/s0140-6736(18)32849-6.

**Table 1**

**Primers utilized for qRT-PCR**

| Target | Primer sequence (5’-3’) |
| --- | --- |
| TBX1 | Forward: CAACAACCTACTGGACGACAACG |
|  | Reverse: CTCCTCGGCATATTTCTCGCTAT |
| GAPDH | Forward: GACCTGACCTGCCGTCTAG |
|  | Reverse: AGGAGTGGGTGTCGCTGT |
| U6 | Forward: GCTTCGGCAGCACATATACT |
|  | Reverse: GTGCAGGGTCCGAGGTATTC |
| miR-6727-5p | Forward: ATCTCGGGGCAGGCGGCT |
|  | Reverse: GCAGGGTCCGAGGTATTC |

Note: qRT-PCR, quantitative real-time PCR; TBX1, T-box transcription factor 1; GAPDH, glyceraldehyde-3-phosphate dehydrogenase; miR-6727-5p, microRNA-6727-5p.

**Table 2**

**Antibodies utilized for Western blot**

| Antibody name | Dilution | Manufacturer | Cat |
| --- | --- | --- | --- |
| p-AKT (Ser473) | 1:1000 | CST | #4060 |
| AKT | 1:2000 | CST | #4691 |
| p-ERK1/2 (Thr202/Tyr204) | 1:1000 | Affinity | AF1015 |
| ERK1/2 | 1:1000 | Affinity | AF0155 |
| p-p38 (Thr180/Tyr182) | 1:500 | Affinity | AF4001 |
| p38 | 1:500 | Affinity | AF6456 |
| p-JNK (Thr183+Tyr185) | 1:1000 | Affinity | AF3318 |
| JNK | 1:2000 | Affinity | AF6318 |
| TBX1 | 1:1000 | Affinity | AF0327 |
| GAPDH | 1:10000 | Proteintech | 60004-1-Ig |
| Goat anti rabbit IgG-HRP | 1:3000 | Solarbio | SE134 |
| Goat anti mouse IgG-HRP | 1:3000 | Solarbio | SE131 |

Note: AKT, protein kinase B; ERK, extracellular signal-regulated kinase; JNK, c-Jun NH2-terminal kinase; TBX1, T-box transcription factor 1; GAPDH, glyceraldehyde-3-phosphate dehydrogenase; IgG, immunoglobulin G; HRP, horseradish peroxidase.

**Figure legends**

**Figure 1 TBX1 expression was decreased in CC tissues and cell lines.** (**A**) TBX1 mRNA expression in CESC tissues and normal tissues. (**B**) Kaplan-Meier plot of TBX1 in patients with CESC. (**C**) Relative mRNA expression of TBX1 was detected by qRT-PCR. CESC, cervical squamous cell carcinoma. * p < 0.05, ** p < 0.01.

**Figure 2 TBX1 inhibited CC cell growth and** **metastasis. (A)** Relative mRNA expression of TBX1 was detected by qRT-PCR. **(B, C)** Cell viability was determined by CCK-8 assay. (**D**) Flow cytometry was used to detect the apoptosis rate of CC cells. (**E**) Cell invasion was detected by transwell assay. Scale bars: 100 μm. **(F, G)** Wound healing assay was performed to determine cell migration ability. Scale bars: 200 μm. * p < 0.05, ** p < 0.01.

**Figure 3 TBX1** **sensitized CC cells to cisplatin*.* (A, B)** CCK-8 assay was used to detect cell viability. **(C)** IC_50_ value was calculated to analyze the cisplatin chemosensitivity of CC cells. **(D)** CC cell apoptosis in the presence of cisplatin (5 μM and 7.5 μM, respectively) for 24 hours was detected by flow cytometry. ** p < 0.01.

**Figure 4 TBX1 inactivated AKT and MAPK signal pathways** **in CC cells.** Western blot was used to evaluate the phosphorylation of proteins related to AKT and MAPK signal pathways in SiHa and Hela cells after transfection with OE-TBX1 plasmids or empty vectors.

**Figure 5 miR-6727-5p targeted and decreased TBX1 expression in SiHa cells. (A)** Potential binding site between miR-6727-5p and TBX1 was predicted using TargetScan database and validated by dual luciferase reporter assay. **(B, C)** Relative expression of miR-6727-5p and TBX1 mRNA were detected by qRT-PCR. **(D)** Expression level of TBX1 protein was detected in SiHa cells by Western blot. * p < 0.05, ** p < 0.01.

**Figure 6** **TBX1 silencing r****eversed the effect of miR-6727-5p inhibitor on SiHa cells.** (**A**) Relative expression level of TBX1 mRNA was detected by qRT-PCR. **(B)** CCK-8 assay was used to measure cell viability. **(C)** Transwell assay was used to detect the invasion capacity of SiHa cells. Scale bar: 100 μm. **(D)** Western blot was adopted to detect the phosphorylation of proteins related to AKT and MAPK signal pathways. ** p < 0.01.
