# Supplementary figures and images for: T-box transcription factor TBX1, targeted by microRNA-6727-5p, inhibits cell growth and enhances cisplatin chemosensitivity of cervical cancer cells through AKT and MAPK pathways
Source: Bioengineered. 2021 Feb 8;12(1):565–77. doi: 10.1080/21655979.2021.1880732 (PMC8806341; doi:10.1080/21655979.2021.1880732)

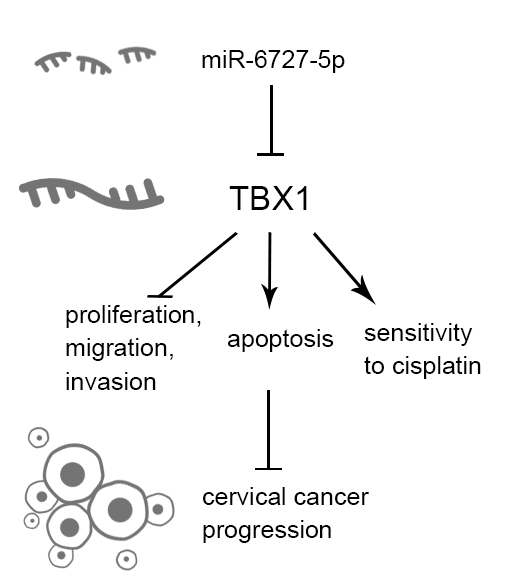

Supplement: Supplemental Material [file KBIE_A_1880732_SM4793.zip › 1880732/Graphical Abstract.tif]
